# Sleep Disturbance and Depression in Older Adults: The Role of Dementia Severity

**DOI:** 10.1093/geroni/igaf122.2924

**Published:** 2025-12-31

**Authors:** Hye Rim Ryu, Cari Cohen, Jerry J Sweet, Christopher Williams, Elizabeth K Geary, Julia Thomas, Leslie Guidotti Breting

**Affiliations:** Rosalind Franklin University of Medicine and Science, Glenview, Illinois, United States; Endeavor Health - NorthShore Hospitals, Chicago, Illinois, United States; Endeavor Health - NorthShore Hospitals, Chicago, Illinois, United States; Endeavor Health - NorthShore Hospitals, Chicago, Illinois, United States; Endeavor Health - NorthShore Hospitals, Chicago, Illinois, United States; Endeavor Health - NorthShore Hospitals, Chicago, Illinois, United States; Endeavor Health - NorthShore Hospitals, Chicago, Illinois, United States

## Abstract

Between 40-70% of older adults in the U.S. report sleep disturbance (Crowley, 2022; Smagula et al., 2016). Poor sleep is common in dementia (Wong & Lovier, 2023) and widely associated with adverse outcomes among older adults, including depression (Li et al., 2022). However, less is known about how the severity of dementia influences this association. The present study thus examined whether dementia severity moderates the relationship between sleep disturbance (gathered from clinical interview) and depressive symptoms (measured by the Geriatric Depression Scale). Participants were older adults with a primary dementia diagnosis following outpatient neuropsychological evaluation (N = 196, Mage = 76.60, SDage = 9.71). Dementia severity was categorized as mild (n = 122), moderate (n = 62), and severe (n = 12). Moderated regression analyses demonstrated that increased sleep disturbance predicted higher depressive symptomatology (B = 6.24, p < .01), with dementia severity moderating this association (p = .04). This moderating relationship was significant among older adults with moderate to severe dementia. Results indicated stronger effects in older adults with severe dementia (B = 10.56, p < .01) compared to those with moderate dementia (B = 6.24, p < .01). Overall, these findings demonstrate that that the association between increased sleep disturbance and higher depressive symptoms becomes stronger as dementia progresses. This study provides further insights into the complex intersection between sleep and mood within the context of neurodegenerative disorders. Results underscore the need for targeted interventions addressing sleep disturbance at varying levels of dementia severity to mitigate depressive symptoms.

